# Cognitive, Psychophysiological, and Perceptual Responses to a Repeated Military-Specific Load Carriage Treadmill Simulation

**DOI:** 10.1177/00187208231214216

**Published:** 2023-11-28

**Authors:** Christopher A. J. Vine, Oliver R. Runswick, Sam D. Blacker, Sarah L. Coakley, Andrew G. Siddall, Stephen D. Myers

**Affiliations:** 1 2476 Institute of Applied Sciences, University of Chichester, UK; 2 4616 King’s College London, UK; 3 62693 St Mary’s University, UK

**Keywords:** soldier, performance, working memory

## Abstract

**Background:**

Dismounted military operations require soldiers to complete cognitive tasks whilst undertaking demanding and repeated physical taskings.

**Objective:**

To assess the effects of repeated fast load carriage bouts on cognitive performance, perceptual responses, and psychophysiological markers.

**Methods:**

Twelve civilian males (age, 28 ± 8 y; stature, 186 ± 6 cm; body mass 84.3 ± 11.1 kg; V̇O_2max_, 51.5 ± 6.4 mL·kg^−1^·min^−1^) completed three ∼65-min bouts of a Fast Load Carriage Protocol (FLCP), each interspersed with a 65-min recovery period, carrying a representative combat load of 25 kg. During each FLCP, cognitive function was assessed using a Shoot/Don’t-Shoot Task (SDST) and a Military-Specific Auditory N-Back Task (MSANT), along with subjective ratings. Additional psychophysiological markers (heart rate variability, salivary cortisol, and dehydroepiandrosterone-sulfate concentrations) were also measured.

**Results:**

A main effect of bout on MSANT combined score metric (*p* < .001, Kendall’s W = 69.084) and for time on the accuracy-speed trade-off parameter of the SDST (*p* = .025, Ѡ^2^ = .024) was evident. These likely changes in cognitive performance were coupled with subjective data indicating that participants perceived that they increased their mental effort to maintain cognitive performance (bout: *p* < .001, Ѡ^2^ = .045; time: *p* < .001, Ѡ^2^ = .232). Changes in HRV and salivary markers were also evident, likely tracking increased stress.

**Conclusion:**

Despite the increase in physiological and psychological stress, cognitive performance was largely maintained; purportedly a result of increased mental effort.

**Application:**

Given the likely increase in dual-task interference in the field environment compared with the laboratory, military commanders should seek approaches to manage cognitive load where possible, to maintain soldier performance.

## Introduction

Military operators complete physically and cognitively demanding tasks simultaneously. Performance decrements in either domain can result in suboptimal performance at best (Martin, McLeod et al., 2019), to injuries or fatalities at worst (Armstrong III et al., 2004; Eddy et al., 2015). Whilst the influence of acute, nonmilitary-specific aerobic exercise on cognitive function is generally well documented (Giles et al., 2019b), relatively little is known regarding the influence of military-specific physical activity on cognitive function (Giles, Hasselquist et al., 2019).

Seminal research by Knapik et al. (1997) investigated the influence of six load-distributions (34, 48, 61 kg back vs. double pack) on cognitive performance following 20-km best-effort marches. Whilst isolated interaction effects were observed for some cognitive parameters, purportedly a result of variability in baseline performance, differences were not observed between different loads or distributions. It has, however, been suggested that a pre-, postphysical task cognitive assessment methodology, may allow sufficient recovery to maintain cognitive performance (Mahoney et al., 2007). This pre- versus posttest approach therefore may not truly reflect the cognitive capabilities of soldiers during strenuous military activity. Instead, within-task assessments may provide more operationally relevant outcomes whilst increasing the granularity of the evidence base, via the increased number of assessment points (Vine et al., 2021).

More recent publications, utilising a dual-tasking approach, have largely focused upon the influence of external load on cognitive performance (e.g., Armstrong et al., 2022; Eddy et al., 2015; Giles, Hasselquist et al., 2019; Kobus et al., 2010); although other factors such as anxiety (Nibbeling et al., 2014) and terrain (Crowell et al., 1999) have also been investigated. The studies investigating the effects of external load have broadly demonstrated a decrease in cognitive performance with increasing load carried. With load being the principal variable manipulated, it is plausible that these observed decrements in cognitive performance could be attributed to differences in work rate as opposed to carrying the load per se (Mahoney et al., 2007). Importantly, cognitive decrements have typically been demonstrated beyond 30 minutes of exercise (Armstrong et al., 2022; Eddy et al., 2015; Giles, Hasselquist et al., 2019; Kobus et al., 2010), which highlights the importance of military-specific research designs given that soldiers operate for extended time periods.

During modern asymmetric warfare soldiers are required to be responsive, reactive, and often complete tasks sequentially; as such, military tasks are rarely completed in isolation. Despite this likely scenario only one study has sought to investigate cognitive performance during repeated bouts of load carriage (Giles, Hasselquist et al., 2019). Operational situations can often also necessitate the requirement for a fast movement speed in combination with lighter loads; to make the physical demands of the load carriage task attainable and sustainable. Whilst the physical demands of an operationally relevant load carriage task utilising faster velocities (>4.8 km·h^−1^) and lighter load masses (<30 kg; termed “Fast Load Carriage Protocol” [FLCP]) have been reported (Vine et al., 2022a), the cognitive repercussions have not been quantified. Critically, these faster velocities of the FLCP resulted in higher work rates compared with the aforementioned studies that observed an attenuation in cognitive performance (Eddy et al., 2015; Giles, Hasselquist et al., 2019; Kobus et al., 2010). Suggesting a decrement in cognitive performance could be apparent during faster load carriage tasks.

Whilst understanding attenuations in cognitive performance during military taskings is of the upmost importance, there is also a need to investigate factors that may explain variance in this performance; for example, through the use of psychophysiological biomarkers such as heart rate variability (HRV) and stress-related hormones (e.g., cortisol and dehydroepiandrosterone-sulfate [DHEA-S]) (Martin, Périard et al., 2019), as well as differences in subjective ratings. Performance in both a working memory task and a continued performance task were strongly associated with HRV groupings in a sample of 53 Norwegian Naval personnel (Hansen et al., 2003). The same research group observed similar HRV relationships in naval cadets, but also demonstrated an association between cortisol levels and cognitive performance (Johnsen et al., 2012). In a study by Shia et al. (2015), prolonged cortisol responses were also negatively associated with working memory, with DHEA-S:cortisol ratio demonstrating the potential to indicate resilient individuals. Whilst caveats to the research exist (e.g., dichotomous HRV groupings), collectively these biomarkers demonstrate potential.

The current study aimed to investigate the effects of repeated fast load carriage tasks on parameters of cognitive performance relevant to military operators. This study also sought to investigate the influence of repeated fast load carriage tasks on psychophysiological biomarkers. It was hypothesised that both time and repeated bouts would negatively affect cognitive performance.

## Methods

### Participants

Twelve physically active males, with no prior military experience, volunteered to participate (age, 28 ± 8 y; stature, 186 ± 6 cm; body mass 84.3 ± 11.1 kg; maximal rate of oxygen uptake [V̇O_2max_], 51.5 ± 6.4 mL·kg^−1^·min^−1^; body fat percentage, 14.0 ± 4.5%). Ethical approval was granted by the Institution’s Research Ethics Committee, with data collected in accordance with the Deceleration of Helsinki.

### Experimental Approach

The study protocol comprised three distinct elements: (1) familiarisation session, (2) two-day baseline data collection, and (3) experimental session. For both laboratory visits, participants were required to have avoided strenuous exercise and caffeine for 24 hours and three hours, respectively, and attend in a hydrated state, having maintained their habitual diet in the lead up to, and between, sessions. For both sessions, participants wore the same sports t-shirt, shorts, and training shoes.

### Cognitive Assessments

The Military-Specific Auditory N-Back Task (MSANT; Vine et al., 2022a) was designed to mimic aspects of coded military radio traffic. The MSANT, comprised of letter pairs, described phonetically using the International Radiotelephony Spelling Alphabet via an audio file. Each letter within a pair was separated by .4 s, and each pair was separated by 2 s. After a random number of letter pairs (3–7 pairs), an audio tone was sounded, and participants were required to identify the pair of letters played two pairs previously (i.e., 2-Back). The audio track was played to participants via headphones, with answers relayed verbally to the research team. Letter stimuli were generated using online speech generation software (www.fromtexttospeech.com) and randomly selected using an online random number generator (Research Randomiser; https://www.randomizer.org/). Each MSANT lasted approximately 5 minutes and required 10 responses.

The Shoot/Don’t-Shoot Task (SDST) is a previously described visual search and inhibition task, whereby 12 possible target locations are displayed on two levels of an urban scene depicting a derelict warehouse (Vine et al., 2022b). The scene was presented to participants on a large high-resolution screen (1920 × 1080 pixels; Panasonic LED TV VIERA TX-42A400B, Osaka, Japan), 2.6 m in front of the individuals walking position on the treadmill. At random time intervals (.5–3 s), either a target (persons adopting a shooting stance) or nontarget (persons with hands up above their head) would appear at a random window. For a target stimulus, a mouse click was required (no locational movement required), whereas no response was required for a nontarget. Stimuli were not of the same spatial frequency due to this not being representative of real-world scenarios; however, stimuli size was standardised. There was a 2:1 ratio between targets and nontargets, with two targets and one nontarget appearing in each location. Participants were instructed to place equal importance on response speed and response accuracy. The SDST recorded using SuperLab 5 software (version 5.05; Cedrus®, San Pedro, USA), with responses collected via a gaming mouse, with 1 ms latency period (Logitech G203, Logitech, Lausanne, Switzerland) which was attached to the side of a replica assault rifle, of correct mass [mouse button positioned adjacent to the trigger location].

### Fast Load Carriage Protocol

The FLCP is a treadmill-based occupationally relevant load carriage task, which was designed by Vine et al., 2022a. It requires participants to carry a representative load of 25 kg (belt webbing [10 kg], body armour [10 kg] and weapon [5 kg]), for 20 minutes at 5.1 km·h^−1^, 40 minutes, at 6.5 km·h^−1^ (1% gradient), and then complete 8 × 9 second bouts of running at 11 km·h^−1^ (3% gradient) with 11 s recovery between. The first 60 minutes of this protocol are designed to represent fast marches undertaken by individuals within the British Army, whilst the repeated shuttles are designed to mimic offence or defensive fire and manoeuvre tasks.

### Familiarisation Session

Participants’ informed consent was taken, along with the completion of a detailed health history questionnaire. Stature, body mass, and body composition were then recorded. Following this, participants completed a 10-min unloaded walking warm-up on a treadmill (HP Cosmos Saturn, HP Cosmos, Germany) before completing a V̇O_2max_ assessment and subsequent verification (same manner as previously described; Draper et al., 2006; Midgley et al., 2009; Vine et al., 2022a).

Following a 15-min recovery period, participants were familiarised with the two cognitive assessments in a seated position. They completed the MSANT and SDST twice, before then proceeding to complete an abridged version (approx. 21-min) of the FLCP. This level of familiarisation has previously been demonstrated to lead to no likely further improvements in performance (Vine et al., 2022b). During the familiarisation and subsequent full FLCP participants wore a belt webbing system, body armour, and a replica assault rifle with sling, totalling ∼25.0 kg. The replica assault rifle was carried in the “ready position” with the weapon slung across their chest and supported by both hands. During each 10-min period participants completed both the MSANT and SDST once.

### Quantification of Baseline Values

In the two days prior to the experimental trial, participants were required to collect, a resting HRV measurement, saliva samples, and complete several questionnaires. This was repeated on the morning of the main trial, to provide a three-day baseline period. Specifically, immediately upon waking, participants were required to don a heart rate (HR) chest belt (Polar v800, Polar Electro, Finland), and follow provided instructions, to commence a 10-min supine HRV measurement. During the HRV measurement, participants were instructed to minimise movement, maintain normal breathing, and avoid any distractions. Immediately upon completion participants were then required to collect a saliva sample using the unstimulated passive drool technique, in the manner described by the assay manufacturer (Salimetrics, Carlsbad, USA). Once complete, participants provided ratings of sleepiness (Karolinska Sleepiness Scale; Åkerstedt & Gillberg, 1990), and fatigue (Samn-Perreli fatigue questionnaire; Samn & Perelli, 1982). Wake up, and subsequent assessment times were based on experimental trial timings and were standardised for both days. In the afternoon of the two baseline days, participants were required to collect a second saliva sample. Again, timings of this collection were in line with the sample time at the end of the experimental trial. For saliva collections, participants recorded the collection time and stored their samples in their home freezers (−20°C).

### Experimental Trial

On the morning of the experimental trial, participants followed the same morning baseline data collection routine, that they had on the previous two days. Participants consumed a standardised breakfast of instant porridge 1 hour before attending the laboratory, having fasted for the previous 12 hours. Upon arrival at the laboratory, participants undertook a standardised five-minute warm-up. A HR monitor was then fitted to the participant, and the load ensemble was donned. Participants then commenced the FLCP.

During the FLCP, HR was recorded continuously, with data averaged across the last minute of each five-minute “block” (Table 1). In alternating “blocks” cognitive performance was assessed with either the MSANT or the SDST. At the end of each five-minute “block” participants were required to provide their RPE (Borg, 1970), Rating Scale of Mental Effort (RSME; (Zijlstra, 1993), and both their thermal sensation and comfort (ASHRAE Standard, 1992; Bedford, 1936). A 150 mL bolus of water was provided to participants at four-time points during the FLCP (Sawka et al., 2007).

**Table 1.** Overview of Experimental Measures and Their Timings During the Fast Load Carriage Protocol.

Measurement	Time (Minutes)													
	0	5	10	15	20	25	30	35	40	45	50	55	60	65^a^
Speed (km·h^−1^)	0	5.1	5.1	5.1	5.1	6.5	6.5	6.5	6.5	6.5	6.5	6.5	6.5	FM
Gradient (%)	0	1	1	1	1	1	1	1	1	1	1	1	1	3
Perceptual Scales	✓	✓	✓	✓	✓	✓	✓	✓	✓	✓	✓	✓	✓	✓
HR		✓	✓	✓	✓	✓	✓	✓	✓	✓	✓	✓	✓	✓
MSANT			✓				✓				✓			
SDST					✓				✓				✓	
Environmental	✓	✓	✓	✓	✓	✓	✓	✓	✓	✓	✓	✓	✓	✓
Water					✓		✓		✓				✓	

*Note.* FM, Fire and Manoeuvre Speeds – see methodology for a detailed description of the treadmill speed in this section of the protocol; HR, heart rate, MSANT, Military-Specific Auditory N-Back Task; SDST, Shoot/Don’t-Shoot task.

^a^Note this block is not 5 minutes in duration – see methodology for a detailed description of the duration of this section of the protocol.

Upon completion of the FLCP, participants took off the additional load mass and moved to a quiet, adjacent room where they rested, prone, to allow for a 10-min HRV measurement. Once completed, participants were provided with a standardised cereal bar and a chocolate milk drink. The macronutrient composition and caloric provision were based on previous field-based data (Ahmed et al., 2019; Edwards, 2020), scaled for the duration of the experimental trial. Participants then rested, in a seated position, until they were required to re-warm-up and commence the next FLCP bout (65-min total inter bout period). Participants completed three iterations of the above-detailed methodology with all protocols remaining consistent, except for iteration three, where they provided a saliva sample before consuming their snack.

### Biochemistry

Baseline saliva samples were brought to the laboratory on the day of the main experimental trial and stored at −20°C. On a separate day, samples were thawed before being centrifuged at 1500 g for 15 minutes and transferred into 2 mL aliquots. Samples were then stored at −80°C*.* Samples were initially thawed at room temperature before being analysed for cortisol and DHEA-S by ELISA in accordance with manufacturer’s guidelines (assay kits 1–3002, and 1–125 respectively; Salimetrics, Carlsbad, USA). Assay controls and samples were analysed in duplicate and on the same plate using a microplate reader (SPECTROstar Nano, BMG Labtech, Aylesbury, UK) and proprietary software (MARS, BMG Labtech, Aylesbury, UK). For comparative purposes, sample concentrations were converted into nmol·L^−1^, utilising correction factors supplied by the assay manufacture. Due to the variance in DHEA-S associated with the salivary drool period, concentrations were corrected for drool time. Intra assay coefficients of variation were 6.1 and 18.5%, respectively.

### Data Analysis

For the MSANT, the variables of correct responses (both letters correctly identified), partially correct responses (one of the two letters correctly identified [in the correct location]), and total combined correct responses ([3 × correct responses] + [1 × partial correct responses]) were calculated. For the SDST, the variables of shoot correct, don’t-shoot correct, total correct (∑ shoot correct + ∑ don’t-shoot correct), average response time, and accuracy-speed trade-off (ASTO; Average response time ÷ Total correct responses were calculated. Heart rate data are reported as a percentage of heart rate reserve (%HRR; [maximum HR during the V̇O_2max_ assessment - minimum HR during supine rest]. Kubios HRV Standard Software (v3.3.1, Kubios, Biosignal Analysis and Medical Imaging Group, Finland) was used for the analysis of HRV data, with a low artefact correction threshold applied. To minimise the influence of prior exercise, analysis occurred for the second 5 minutes of the measurement period. The key variables of average RR interval, HR, root mean square of the successive differences (RMSSD), High-Frequency (HF) Power, and Low-Frequency (LF) Power are reported.

### Statistical Analysis

Statistical analysis was conducted using JASP (v0.11.1, University of Amsterdam, Netherlands), with data presented as mean ± standard deviation. Using base-2 log transformations of *p*-values, S-values (Shannon, 1948) were calculated to aid clarity and interpretation of statistical estimation (Cole et al., 2021). Data normality were assessed using skewness and kurtosis ratios, with sphericity also assessed. The Greenhouse-Geisser correction was applied if assumptions of sphericity were violated. For, HRV-, cortisol- and DHEA-S-derived variables, a one-way ANOVA for time was run, whilst for all other investigated variables a two-way repeated-measures ANOVA was employed to investigate time, FLCP bout, and interaction effects. Omega squared (Ѡ^2^) effect sizes are presented (Levine & Hullett, 2002). For nonnormally distributed data, a Friedman’s test was employed with effect sizes presented using Kendall’s W. Where test statistics, *p*-values/S-values, and effect sizes indicated a likely incompatibility with the null model, *post-hoc* pairwise comparisons, with a Holm-Bonferroni adjustment (denoted by a subscript H), were made. These comparisons are presented as mean differences ± Bonferroni adjusted 95% compatibility (confidence) intervals. For *post-hoc* comparisons, Cohen’s standardised means effect sizes were calculated and converted to Hedge’s gz (Lakens, 2013), to adjust for the overestimate of effect sizes associated with small sample sizes. For instances where multiple differences are observed, ranges of *p*-values and effect sizes are presented. For nonnormally distributed data *post-hoc* pairwise comparisons were made using Conover’s test.

## Results

Across the three FLCP bouts, environmental conditions remained consistent (median bout average 13.2 ± 0.8°C WBGTi, 57 ± 5% relative humidity). Baseline sleep and fatigue questionnaires indicated participants consistently deemed on average they were “A little tired, less than fresh” and “Neither alert nor sleepy” following the three nights preceding the experimental trial.

### Physiological and Perceptual Responses

**Figure 1.**
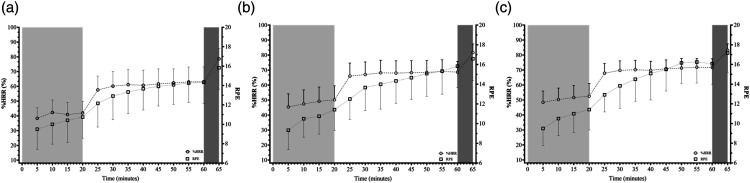
Percentage Heart Rate Reserve (%HRR) and Ratings of Perceived Exertion (RPE) data for bout 1 (a), bout 2 (b), and bout 3 (c) of the Fast Load Carriage Protocol. *Note.* Light grey, white, and dark grey areas denote the 5.1 km·h^−1^, 6.5 km·h^−1^, and simulated fire and manoeuvre portions of the protocol, respectively.

**Figure 2.**
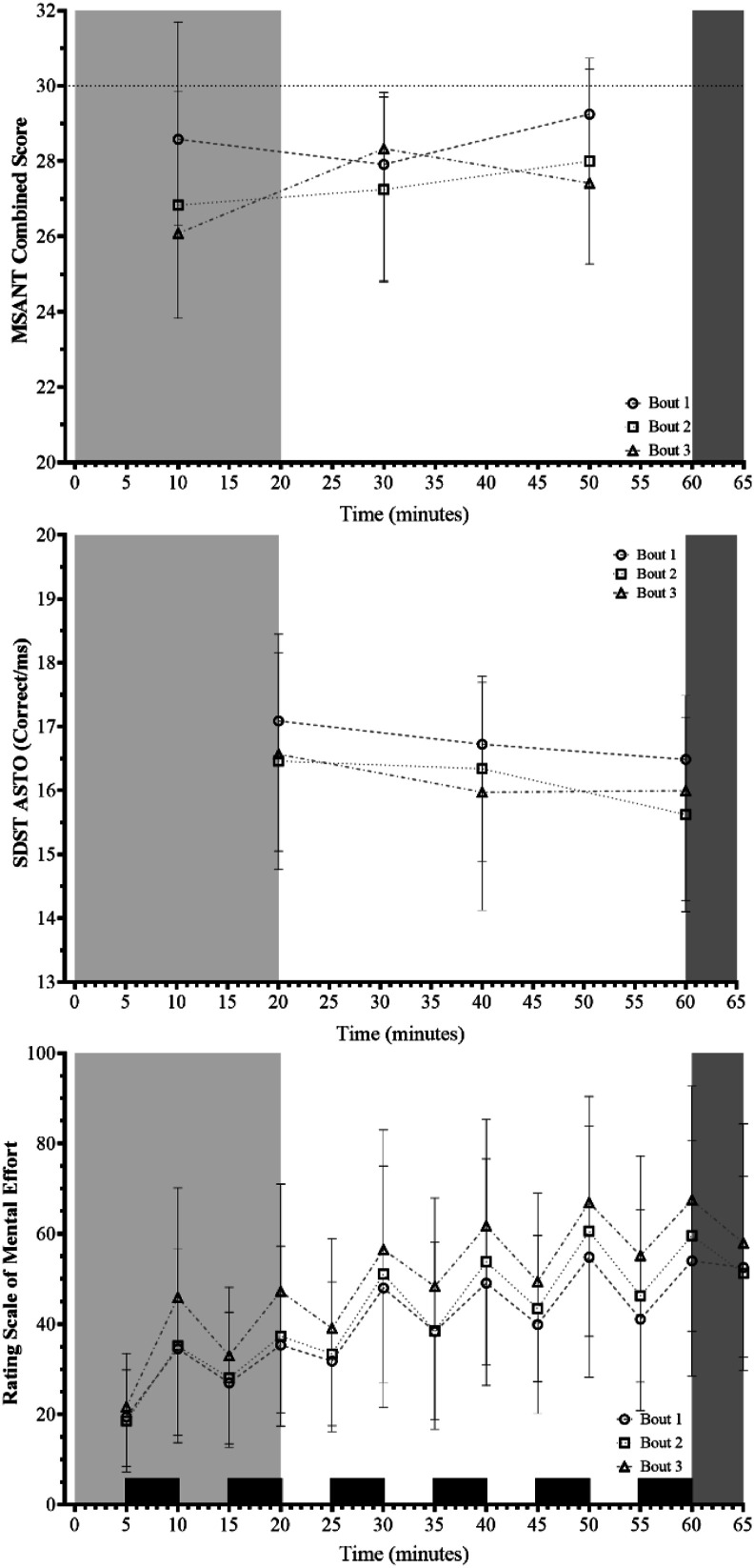
Military Specific Auditory N-back Task (MSANT) combined score, Shoot/Don’t-Shoot Task (SDST) Accuracy-Speed Trade-Off (ASTO) score, and Rating Scale of Mental Effort (RMSE) data during the three bouts of the Fast Load Carriage Protocol. *Note.* Light grey, white, and dark grey areas denote the 5.1 km·h^−1^, 6.5 km·h^−1^, and simulated fire and manoeuvre portions of the protocol, respectively. Circle, square, and triangle symbols denote data for bout 1, 2, and 3, respectively. Dotted line on top panel denotes maximum combined score.

Physiological strain, normalised for each participant, as described by %HRR, in combination with RPE data, is shown in Figure 1, whilst RSME is shown alongside cognitive performance data in Figure 2. The %HRR data demonstrated a likely effect for both bout and time (bout: *F*_(2, 22)_ = 50.409, *p* < .001, S > 9.97, Ѡ^2^ = .195; time: *F*_(11, 121)_ = 544.603, *p* < .001, S > 9.97, Ѡ^2^ = .593), but did not suggest an interaction effect was present (*F*_(22, 121)_ = 1.044, *p* = .411, S = 1.28, Ѡ^2^ = 1.398e^-4^). Average HR across the 20 minutes at 5.1 km·h^−1^ was 105 ± 16, 115 ± 18, and 118 ± 16 beats·minute^−1^ for bouts 1, 2, and 3, respectively; whilst average HR across the 40 minutes at 6.5 km·h^−1^ was 133 ± 19, 143 ± 17, and 146 ± 15 beats·minute^−1^ for bouts 1, 2, and 3, respectively. For bouts 1, 2, and 3 peak HR during the shuttles was 157 ± 16, 161 ± 14, and 164 ± 14 beats·minute^−1^ respectively. The RPE data demonstrated a main effect of bout, time, and a bout-time interaction effect (bout: *F*_(2, 22)_ = 7.873, *p* = .003, S = 8.38, Ѡ^2^ = .047; time: *F*_(11, 121)_ = 377.726, *p* < .001, S >9.97, Ѡ^2^ = .280; interaction: *F*_(22, 121)_ = 168.492, *p* < .001, S >9.97, Ѡ^2^ = .221). Similarly, the RSME data differed for both bout and time (bout: *F*_(2, 20)_ = 20.546, *p* < .001, S >9.97, Ѡ^2^ = .045; time: *F*_(12, 120)_ = 14.851, *p* < .001, S >9.97, Ѡ^2^ = .232); but a bout-time interaction was unlikely (*F*_(24, 240)_ = 1.164 *p* = .277, S = 1.85, Ѡ^2^ = 9.391e^-4^). At the start of the trials, participants were indicating a requirement for “almost no [mental] effort”, however, by the end of 60 minutes, participants reported having to make “considerable [mental] effort” to complete their required tasks. Figure 2 shows that the RSME scores oscillated, with ratings higher following the completion of the cognitive assessments.

### Cognitive Performance Measures

Data for the key cognitive performance parameters are listed in Table 2, with principal cognitive outputs illustrated in Figure 2. For the combined score metric of the MSANT, a likely difference was evident for bout, but not time (bout: χ^2^_(2)_ = 7.154, *p* < .001, S >9.97, Kendall’s W = 69.084; time: χ^2^_(2)_ = 3.581, *p* = .083, S = 3.59, Kendall’s W = 63.277). However, following *post-hoc* comparisons, the location of these bout differences was not apparent (*p*_*H*_*’s* = 1.00, S’s = .00). For the SDST, an effect for time was likely for ASTO (*F*_(2, 22)_ = 4.395 *p* = .025, S = 5.32, Ѡ^2^ = .024). Conversely, statistical analysis did not provide evidence for a likely bout (*F*_(2, 22)_ = 1.808 *p* = .188, S = 2.41, Ѡ^2^ = .013) or an interaction effect (*F*_(4,44)_ = .318 *p* = .865, S = .21, Ѡ^2^ = .000). *Post-hoc* comparisons, suggested a likely difference between time points 1 and 3 (*t*_(2)_ = 2.962, *p*_*H*_ = .022, S_*H*_ = 5.51, *g*_*z*_ = .795, 95% CI_*H*_ [.084, 1.257]), but not between time points 1 and 2, (*t*_(2)_ = 1.59, *p*_*H*_ = .252, S_*H*_ = 1.99, *g*_*z*_ = .427, 95% CI_*H*_ [−.226, .947]), or 2 and 3 (*t*_(2)_ = 1.371, *p*_*H*_ = .252, S_*H*_ = 1.99, *g*_*z*_ = −.368, 95% CI_*H*_ [-.276, .897]).

**Table 2.** Cognitive Performance Parameters During the Three Bouts of the Fast Load Carriage Protocol.

		Median and [Range] Performance Scores at Each Measurement Point^a^								
		Bout 1			Bout 2			Bout 3		
		Time Point 1	Time Point 2	Time Point 3	Time Point 1	Time Point 2	Time Point 3	Time Point 1	Time Point 2	Time Point 3
MSANT	Correct responses	10 [7–10]	9 [5–10]	10 [9–10]	9 [6–10]	9 [7–10]	10 [7–10]	9 [2–10]	9 [8–10]	9.5 [6–10]
	Partially correct responses	0 [0–3]	.5 [0–4]	0 [0–1]	.5 [0–3]	1 [0–2]	0 [0–3]	.5 [0–4]	1 [0–1]	0 [0–4]
	Combined score	30 [24–30]	28 [19–30]	30 [27–30]	27 [21–30]	27.5 [23–30]	30 [23–30]	27.5 [10–30]	28 [25–30]	28.5 [22–30]
SDST	Shoot correct responses	24 [23–24]	24 [23–24]	24 [20–24]	24 [22–24]	24 [20–24]	24 [24–24]	24 [23–24]	24 [24–24]	24 [23–24]
	Don’t-shoot correct responses	11 [9–12]	12 [4–12]	12 [10–12]	11 [10–12]	11 [10–12]	12 [10–12]	12 [9–12]	11 [10–12]	11 [8–12]
	Response time (ms)	608 [484–672]	557 [469–702]	571 [478–717]	591 [476–650]	569 [491–630]	555 [484–642]	595 [461–691]	556 [488–652]	569 [472–636]
	ASTO (total correct responses·ms^−1^)	17.1 [13.5–20.4]	16.6 [13.0–20.8]	16.5 [13.3–20.5]	16.7 [13.2 – 19.0]	16.2 [14.0 – 18.0]	15.6 [13.5–18.3]	17.0 [12.8–20.3]	15.5 [13.9 – 19.0]	16.3 [13.1–18.2]

*Note.* MSANT, Military Specific Auditory N-back Task; SDST, Shoot/Don’t-Shoot Task; ASTO, Accuracy-Speed Trade-Off score.

^a^Time points for each cognitive assessment are not time aligned, see methods section for exact timings of cognitive assessments.

### Psychophysiological Measures

**Figure 3.**
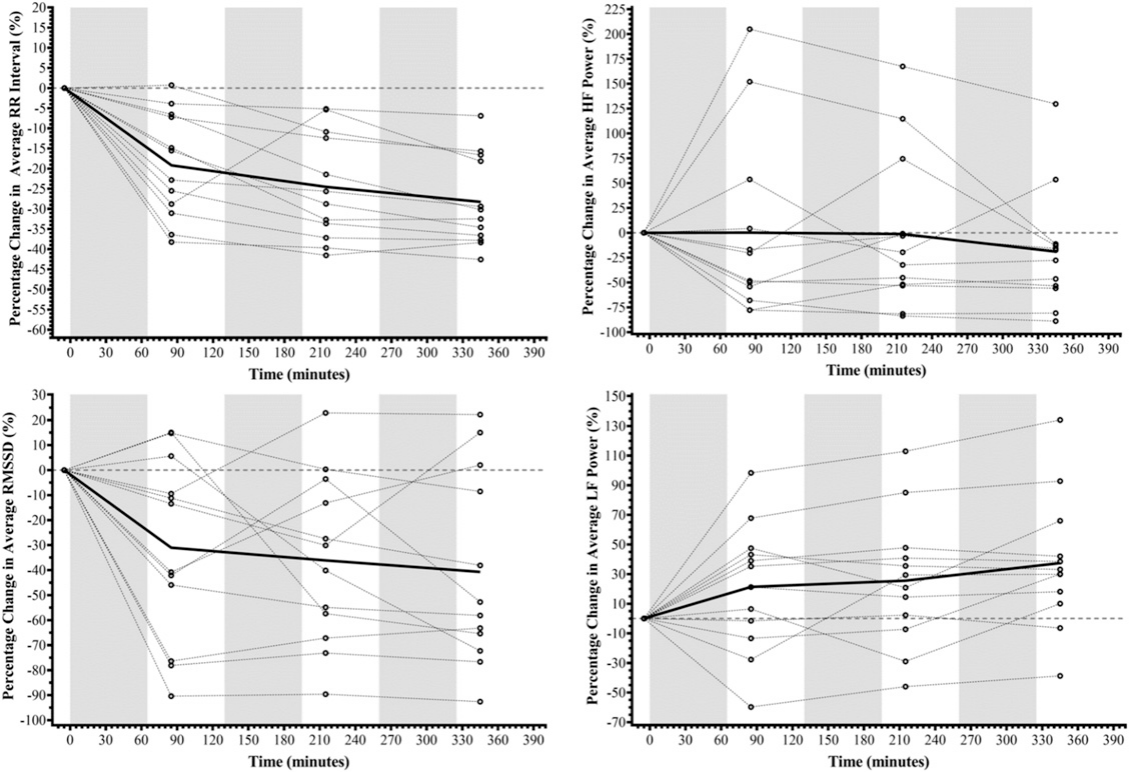
The percentage change in average RR interval, Root Mean Square of the Successive Differences (RMSSD), High Frequency (HF) Power, and Low Frequency (LF) Power across the three Fast Load Carriage Protocol bouts. *Note.* Black circles (o) denote individual data points, with dotted lines connecting these across assessment points; thick black line denotes the mean average for the group across assessment points; greyed areas denote each of the three Fast Load Carriage Protocols completed.

Figure 3 displays HRV data. When compared to the average values for the three-day baseline period, there was evidence of a time effect for average RR interval, average HR, RMSSD, and LF power, but not HF power (RR interval: *F*_(1.878,20.659)_ = 28.612, *p* < .001, S >9.97, Ѡ^2^ = .248; average HR: *F*_(1.902,20.922)_ = 23.039, *p* < .001, S >9.97, Ѡ^2^ = .215; RMSSD: *F*_(1.756,19.313)_ = 5.982, *p* = .012, S = 6.38, Ѡ^2^ = .051; LF power: *F*_(3, 33)_ = 3.867, *p* = .018, S = 5.80, Ѡ^2^ = .057; HF power: *F*_(3, 33)_ = 1.173, *p* = .335, S = 1.58, Ѡ^2^ = .004). Following the first bout of load carriage, average RR interval decreased 19% (*t*_(3)_ = 5.691, *p*_*H*_ < .001, S_*H*_ >9.97, *g*_*z*_ = 1.528, 95% CI_*H*_ [112.086, 330.216]), resulting in a 26% increase in average HR (*t*_(3)_ = -6.188, *p*_*H*_ < .001, S_*H*_ >9.97, *g*_*z*_ = −1.662, 95% CI_*H*_ [-22.449, 6.188]). This trend continued across all three bouts with 28% decreases in average RR interval and 42% increase in average HR following the third bout (average RR interval: *t*_(3)_ = 8.512, *p*_*H*_ < .001, S_*H*_ >9.97, *g*_*z*_ = 2.286, 95% CI_*H*_ [221.713, 439.843]; average HR: *t*_(3)_ = −7.688, *p*_*H*_ < .001, S_*H*_ >9.97, *g*_*z*_ = −2.064, 95% CI_*H*_ [−30.399, −14.139]). Decreases of 31, 36 and 41% were observed on average for RMSSD values following the first, second and third bout of FLCP, respectively (first: *t*_(3)_ = 3.013, *p*_*H*_ = .020, S_*H*_ = 5.64, *g*_*z*_ = .809, 95% CI_*H*_ [1.480, 41.835]); second: *t*_(3)_ = 3.243, *p*_*H*_ *=* .014, S_*H*_ = 6.16, *g*_*z*_ = .871, 95% CI_*H*_ [3.133, 43.488]); third: *t*_(3)_ = 3.883, *p*_*H*_ = .003, S_*H*_ = 8.38, *g*_*z*_ = 1.043, 95% CI_*H*_ [7.739, 48.094]).

### Biochemical Markers

**Figure 4.**
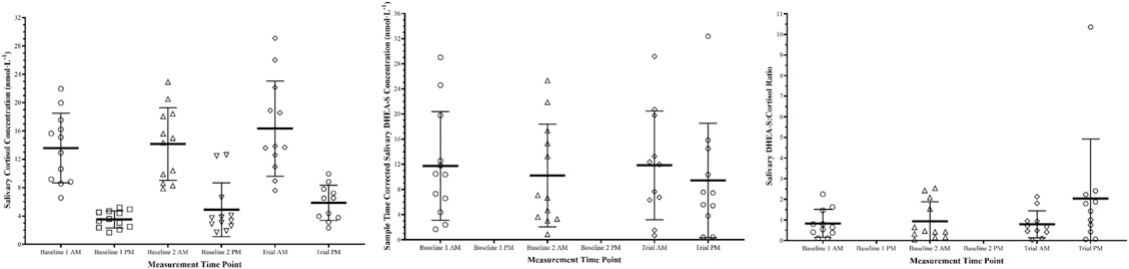
Saliva Biomarker Responses to three Fast Load Carriage Protocol bouts compared with a baseline period. *Note.* Baseline 1 is two days prior to experimental trial, baseline 2 is one day prior to trial. Note DHEA-S was not measured at baseline PM samples, and therefore not presented on the middle and right figures. Thicker line denotes data mean average, and tails denote one standard deviation.

Salivary cortisol and DHEA-S data are shown in Figure 4. When DHEA-S concentrations were expressed relative to cortisol concentrations a main effect of measurement point appeared evident (*F*_(3,27)_ = 4.169, *p* = .015, S = 6.16, Ѡ^2^ = .091). *Post-hoc* comparisons provided evidence that this ratio was greater for the final measurement point compared to all three previous measurement points (Baseline 1 AM: *t*_(3)_ = −2.718, *p*_*H*_ *=* .045, S_*H*_ = 4.47, *g*_*z*_ = −.730, 95% CI_*H*_ [−1.060–.025]; Baseline 2 AM: *t*_(3)_ = −3.020, *p*_*H*_ *=* .033, S_*H*_ = 4.92, *g*_*z*_ = −.811, 95% CI_*H*_ [−1.117 to −.033]; Trial AM: *t*_(3)_ = −2.893, *p*_*H*_ *=* .037, S_*H*_ = 4.76, *g*_*z*_ = −.777, 95% CI_*H*_ −1.093–−.009]).

## Discussion

The present study assessed the impact of repeated bouts of military-specific physical activity on cognitive performance relating to operational requirements. Results demonstrate an elevated physiological strain for each successive bout of load carriage, reflected in HR and perceptual ratings. Critically, despite the increase in physiological and psychological stress, effectiveness of cognitive performance was largely maintained but at the cost of a decrease in cognitive efficiency evidenced through increased RSME ratings and the buffering of the cortisol response by DHEA-S (DHEA-S:cortisol ratio).

The SDST analysis suggested there was a progressive improvement over the duration of the FLCP. The combined score metric of the MSANT demonstrated a likely main effect for bout. Further analysis did not provide evidence of where a difference was apparent; although observationally, each FLCP bout demonstrated a performance reduction. Of the SDST variables, the ASTO is arguably the most critical variable for the military end-user given the equal importance on both accuracy (score) and response time (Vine et al., 2021). Importantly, in order to obtain this performance, participants were required to employ increasingly greater mental effort as the FLCP went on, as indicated by RSME data. Other studies have reported mixed results in choice-reaction and working-memory-based tasks. For example, Eddy et al., (2015) reported no effect of time or load, but did suggest choice response time was slower in the second hour of load carriage. Conversely, both Armstrong et al. (2022) and Kobus et al. (2010) reported no effect of load or time on choice response time, but did indicate a decrease in SDST accuracy. Interestingly, both Armstrong et al. (2022) and Kobus et al. (2010) reported participants adopting a more forward-leaning stance during the heavier load carriage conditions, plausibly limiting their field of vision and in turn affecting their performances. With differences apparent between test modalities and investigations, this highlights the importance of employing a dual-tasking methodology to give greater granuality to the evidence base.

Associations between HRV and cortisol and DHEA-S (and their ratio to each other) have also been highlighted as promising approaches in the understanding of stress responses and changes in cognitive the performance within military operators. (An et al., 2020; Hansen et al., 2003; Haufler et al., 2018; Johnsen et al., 2012; Martin, McLeod et al., 2019; Morgan III et al., 2004, 2009; Rensberger, 2018; Taylor et al., 2007). Data from the current investigation supports this notion, with a considerable decrease in RMSSD, (31, 36 and 41%) compared with baseline values after each FLCP bout. Additionally, there was evidence for an increased DHEA-S:cortisol ratio post the third FLCP bout. Despite an increase in physiological and psychological stress, as evidenced through increased HR and decreased HRV, cognitive performance was largely maintained. Purportedly this could be a result of both increased mental effort (as evidenced by RSME data), and the buffering of the cortisol response by DHEA-S (DHEA-S:cortisol ratio). This neuroprotective role of DHEA-S would be critical within high stress military contexts, given the importance of rapid and accurate decision making and information processing (Shia et al., 2015). It should be acknowledged that the magnitude of DHEA-S intraassay variability (18.5%) places a large caveat on these data and that more data in this area are needed to verify findings.

Within military and occupational settings, an additional factor differentiating soldiers from sporting contexts is the comfort of external load mass carried (Kobus et al., 2010). The current study demonstrated a progressive increase in perceived physical exertion (RPE). We have also previously demonstrated an increase in perceived discomfort from the environmental conditions over the time course of the FLCP (Vine et al., 2022a). Collectively, this combined discomfort, from both the workrate and load carried, would likely increase cognitive load and decrease efficiency of cognitive performance. This notion is supported by the observed increase in RMSE values over the time course of the FLCP bout, and across the three successive FLCP bouts. Notably, when participants were required to undertake a cognitive assessment, further mental effort was required to complete the tasks. These observations suggest that soldiers would have less capacity for conducting other tasks and lends further support to the importance of perceptual data during military taskings.

The principal limitation of the current study was the recruitment of an all-male civilian population. In line with the approach previously discussed (Vine et al., 2021), the thorough test familiarisation, a study population with similar physical characteristics to military operators, and utilisation of externally valid assessment tools were used in an attempt to mitigate the lack of military experience. It is also likely that the controlled nature of the laboratory environment resulted in limited dual task interference effects. In an operational environment these interferences may take a multitude of forms including navigating rough and uneven terrain, and maintaining the required marching pace. Giles, Hasselquist et al. (2019) speculated that these were key factors behind why their findings were more pronounced than those reported from similar laboratory-based studies. Future research should look to address this discrepancy between laboratory and field-based collections, through the addition of dual-task interferences that would be typical in the theatre of operations (e.g., traversing terrain/avoiding obstacles).

In conclusion, the current study has demonstrated that despite the increase in physiological and psychological stress, cognitive performance was largely maintained during repeated load carriage bouts; purportedly a result of increased mental effort and cortisol buffering by DHEA-S. Future investigations should seek to elucidate cognitive performance management strategies for soldiers in situations of greater external stress.

## Key Points

- Despite the increase in physiological and psychological stress, cognitive performance was largely maintained during repeated load carriage bouts.
- A decrease in cognitive efficiency was likely as indicated by the increase in mental effort for a similar level of cognitive performance.
- Likely less favourable changes in HRV parameters, compared to baseline, were progressively observed following each bout of load carriage.
